# Both Canonical and Non-Canonical Wnt Signaling Independently Promote Stem Cell Growth in Mammospheres

**DOI:** 10.1371/journal.pone.0101800

**Published:** 2014-07-14

**Authors:** Alexander M. Many, Anthony M. C. Brown

**Affiliations:** Department of Cell & Developmental Biology, Weill Cornell Medical College, New York, New York, United States of America; Baylor College of Medicine, United States of America

## Abstract

The characterization of mammary stem cells, and signals that regulate their behavior, is of central importance in understanding developmental changes in the mammary gland and possibly for targeting stem-like cells in breast cancer. The canonical Wnt/β-catenin pathway is a signaling mechanism associated with maintenance of self-renewing stem cells in many tissues, including mammary epithelium, and can be oncogenic when deregulated. Wnt1 and Wnt3a are examples of ligands that activate the canonical pathway. Other Wnt ligands, such as Wnt5a, typically signal via non-canonical, β-catenin-independent, pathways that in some cases can antagonize canonical signaling. Since the role of non-canonical Wnt signaling in stem cell regulation is not well characterized, we set out to investigate this using mammosphere formation assays that reflect and quantify stem cell properties. Ex vivo mammosphere cultures were established from both wild-type and *Wnt1* transgenic mice and were analyzed in response to manipulation of both canonical and non-canonical Wnt signaling. An increased level of mammosphere formation was observed in cultures derived from MMTV-*Wnt1* versus wild-type animals, and this was blocked by treatment with Dkk1, a selective inhibitor of canonical Wnt signaling. Consistent with this, we found that a single dose of recombinant Wnt3a was sufficient to increase mammosphere formation in wild-type cultures. Surprisingly, we found that Wnt5a also increased mammosphere formation in these assays. We confirmed that this was not caused by an increase in canonical Wnt/β-catenin signaling but was instead mediated by non-canonical Wnt signals requiring the receptor tyrosine kinase Ror2 and activity of the Jun N-terminal kinase, JNK. We conclude that both canonical and non-canonical Wnt signals have positive effects promoting stem cell activity in mammosphere assays and that they do so via independent signaling mechanisms.

## Introduction

Stem cells of the adult mammary gland are predicted to have a capacity for self-renewal and to give rise to the two major epithelial cell lineages of mammary ducts: luminal and basal. Substantial progress has been made towards characterizing mouse mammary stem cell populations, both *in vivo* and *in vitro*, but much remains to be determined about the signaling pathways that regulate their behavior. Elucidating the relevant mechanisms is important for understanding normal stem cell and tissue biology, and also because of the potential for developing therapies that can target stem-like cells in cancer.

Evidence that adult mammary tissue contains multipotent self-renewing stem cells was first provided by classical transplantation studies in which a normal epithelial ductal tree, comprising both basal and luminal cell lineages, could be regenerated from small tissue fragments or individual cells [1], [2]. Such assays were subsequently used prospectively to identify several combinations of surface markers that enrich for cells with mammary repopulating activity, and indicated that stem cells were distributed within the basal epithelial layer [3], [4]. More recently, however, *in vivo* lineage tracing experiments have challenged some of these conclusions [5], [6], suggesting that much of the post-natal development of mammary epithelium is dependent on separate luminal and basal progenitors acting in combination with a smaller population of bipotent stem cells [5]–[7].

*Ex vivo* assays of mammary epithelial cell sphere formation in suspension culture, mammospheres, offer a complementary approach to stem cell studies that is amenable to signaling pathway analysis. Originally developed for analysis of neuronal precursors, the ability of cells to form spheroids has been used as a stem cell assay for several other tissue types, including prostate and mesenchymal stem cells [8]–[11]. Mammosphere-forming cell cultures exhibit stem cell properties in their capacity to self-renew and ability to differentiate into committed luminal and basal lineages [12]. In addition, the ability to form mammospheres correlates with the potential to generate epithelial ductal trees in mammary reconstitution assays [13], [14]. Thus, mammosphere formation has been used as an indicator of cells with stem cell properties in mouse and human mammary cell lines as well as in primary tissue culture [13]–[19].

The canonical Wnt/β-catenin signaling pathway is one of the principal signaling mechanisms associated with regulation of stem cell behavior in numerous tissues [20]–[23]. Canonical Wnt signaling also has well established roles in regulating embryonic development and adult tissue homeostasis, where many of its functions may result from effects on stem or progenitor cells [20]–[22], [24], [25]. Similarly, the Wnt/β-catenin pathway is frequently activated in a wide range of human cancers and may regulate neoplasia in part via modulation of cancer cells with stem-like cell properties [22], [23], [26].

The MMTV-*Wnt1* mouse strain is a well characterized model for the studying the consequences of Wnt signaling in the mammary gland and its effects on stem cells [27]–[30]. The mouse mammary tumor virus (MMTV) promoter drives expression of the *Wnt1* transgene predominantly in luminal epithelium, and results in activation of canonical Wnt/β-catenin signaling in the basal layer [31]–[33]. MMTV-*Wnt1* mice display widespread mammary epithelial hyperplasia and are predisposed to carcinomas with nearly 100% penetrance [34]. Notably, the pre-cancerous hyperplastic tissue of MMTV-*Wnt1* was reported to contain larger numbers of CD24^+^ CD29^HI^ cells, which are enriched for stem cell activity, in comparison to wild-type glands [3]. In a matrigel-based colony assay, wild-type mouse mammary cells selected for the CD24^+^ CD29^HI^ immunophenotype showed increased colony formation in response to purified Wnt3a [35]. Moreover, lineage tracing experiments using Cre-mediated recombination to mark the descendants of Wnt/β-catenin responsive cells expressing Axin2 suggest that such cells contribute to a stem cell population [36]. Collectively these data support a role for Wnt/β-catenin signaling in the growth and/or maintenance of mammary stem cells.

Intracellular signaling elicited by members of the Wnt family of secreted ligands can be broadly classified into two modes: canonical and non-canonical. In the canonical pathway, Wnt ligand binding to receptor complexes containing Frizzled and Lrp5/6 proteins results in stabilization of cytoplasmic β-catenin and transcriptional activation mediated by β-catenin/TCF complexes [22], [37]. Wnt1 and Wnt3a are prototypical examples of ligands that consistently activate this pathway [38]. In contrast, non-canonical Wnt signaling is defined as a signaling response to Wnt ligands that is independent of β-catenin stabilization [39], [40]. Wnt5a exemplifies a Wnt protein that typically signals in a non-canonical manner [38], [41]. Several non-canonical signaling pathways have been proposed and the cognate receptors include Frizzled proteins, and receptor tyrosine kinases such as Ror2, while Lrp5/6 are not required for non-canonical signaling [41]–[44].

While there is strong support for Wnt/β-catenin signaling promoting mammary stem cell properties, as described above, the roles of non-canonical signaling in the mammary gland are much less clear. Moreover, apparently conflicting data exists concerning functional interactions between canonical and non-canonical Wnt signaling in other experimental systems. Thus Wnt5a has been reported to act either act in opposition to, in concert with, or independently of Wnt/β-catenin signaling [45]. In the mouse mammary gland, Wnt5a overexpression has been shown to inhibit ductal extension during development, and to reduce the growth rate of certain tumors [46], [47]. However, due to the multiplicity of non-canonical signaling pathways that Wnt5a may activate depending on the context, it is essential to test its functional consequences empirically in individual assays.

To elucidate the effects of canonical and non-canonical Wnt signaling on stem cell properties of mouse mammary epithelium, here we test the consequence of altered Wnt signaling activity on mammosphere cultures, specifically quantifying the number of secondary mammosphere-forming units (MFUs)[12], [13], [48]. To include all potential stem cells, including those that may not express the cell surface markers previously used for enrichment, we used unsorted populations of mammary epithelial cells [3], [4]. Contrasting the ability of Wnt5a to antagonize canonical Wnt signaling in other systems, we observed that both Wnt3a and Wnt5a promoted mammosphere formation through distinct signaling pathways. Thus both canonical and non-canonical Wnt signaling have independent abilities to promote stem cell capacity.

## Methods

### Cell culture

Mammospheres culture methods were adapted from Dontu *et al*. [12]. Number 3, 4, 8, and 9 mammary glands were resected from adult mice between 3 and 9 months of age, from a FVB/NJ background. Glands were mechanically minced with a razor blade and digested at 37°C with collagenase (≥250 units/ml) in DMEM/F12 medium for 3 hours with vortexing and pipetting every 30 minutes. The digested tissue was centrifuged at 650×g for 5 minutes. The floating fat layer was removed by aspiration, and the tissue homogenate was digested in 1 mg/ml dispase in DMEM/F12 media with constant pipetting for 3 minutes to generate a single cell suspension. This was washed twice in “mammosphere medium” (DMEM/F12, 20 ng/ml bFGF, 20 ng/ml EGF, 4 µg/ml Heparin, B-27 Supplement, and 1% Penicillin/Streptomycin antibiotic solution) [12] and resuspended in that medium. Remaining clumps of cells were removed by filtration through a 40 µM cell strainer. The cell suspension was enriched for epithelial cells using an Easy-Sep mouse mammary epithelial cell enrichment kit (Stem Cell Technologies) according to manufacturer's instructions. Cells were resuspended in mammosphere medium and plated into 96 well low adherence plates (Corning Costar). Primary mammosphere cultures were fed once by addition of fresh mammosphere medium and the pooled cultures harvested by mild centrifugation and resuspension for assays of secondary sphere formation as described below.

### Secondary mammosphere assay

Primary mammosphere cultures were disassociated with trypsin for 30 minutes in the presence of a vital cell labeling dye, (Di-I, or Cell Tracker Red, Invitrogen). Cell suspensions were filtered through a 40 µM cell strainer and then diluted in mammosphere medium with 1% methylcellulose to limit cell aggregation, and 10 nM dexamethasone to maintain transcription from the MMTV promoter. Cells were plated in 96-well low adherence plates using 24 wells per treatment, and were scored for mammosphere formation after one week of growth. In all secondary mammosphere assays, cells were plated at 1000 cells per well except in lentiviral shRNA knockdown experiments, in which they were plated at 2000 cells per well. All exogenous treatments were given as a single dose to the dissociated cells at the time of secondary assay plating. Mammospheres were defined as colonies in methylcellulose suspension culture that contained 10 or more cells visible under phase contrast, of which fewer than 50% still retained the cell tracking dye, consistent with the dye being diluted out upon successive cell divisions.

### Immunostaining

Secondary mammosphere cultures were harvested and washed with PBS. Mammospheres were fixed and permeabilized with a 1∶1 acetone:methanol fixation solution for 30 minutes at −20°C, and PBS was used to rehydrate the mammospheres for 10 minutes at room temperature. Non-specific antibody binding was blocked with a 3% BSA/PBS solution for 1 hour at room temperature. Primary antibodies to cytokeratin 8 (K8; Troma I antibody, developmental studies hybridoma bank, University of Iowa), and cytokeratin 14 (K14; Abcam, catalog #7800), both at a 1∶200 dilution in 3% BSA/PBS, were applied overnight at 4°C. Mammospheres were then washed twice in PBS. Cells were incubated in a 1∶1000 dilution of Alexafluor-conjugated secondary antibodies (Life technologies) in 3% BSA/PBS for 4 hours. Cells were washed twice with PBS and mounted in Vectashield mounting media (Vector laboratories) including 0.1 µg/ml DAPI.

### Mammosphere assay statistical analysis

Numbers of mammospheres reported are the mean of 24 wells per treatment from representative experiments. All experiments were repeated 3 or more times, demonstrating consistent statistical relationship patterns. Comparisons between treatments were made using Student's t test with p<.05 required for significance.

### Mammosphere samples for qRT-PCR

Mammospheres were prepared as for secondary mammosphere assays, except cells were plated in 6-well low adherence plates. After one week cells were harvested by centrifugation and total RNA was extracted using RNeasy mini kits (Qiagen). cDNA was produced using the iScript cDNA synthesis kit (Bio-Rad). Quantitech primers (Qiagen) were used with SYBR-Green mastermix (Quanta) for quantitative PCR using an MJ Opticon2 system (BioRad) according to manufacturer's instructions.

### Viral infection

To infect mammosphere cultures with lentivirus, primary mammospheres were disassociated as for secondary mammosphere assays. Before plating, single cell suspensions in DMEM/F12 media were mixed with lentiviral particles in PBS at a multiplicity of infection of greater than 5∶1, plus a 1∶200 dilution of Transdux reagent (System Biosciences) for 30 minutes at 37°C. After infection, the cell suspension was plated as for secondary sphere assays. The 7TGC lentiviral reporter vector was obtained from Addgene (Plasmid#24304) [49]. Lentiviral vectors for knockdown of *Ror2* mRNA were obtained from Dr. T. Stappenbeck [50]. For clarity we renamed the knockdown vector shRor2#7 [50] as shRor2 and the control vector SCH002-EGFP as shControl.

### Recombinant proteins and small molecule inhibitors

In experiments using inhibitors and recombinant proteins in conjunction with secondary mammosphere assays, agents were added to single cell suspensions before plating. Recombinant Wnt3a (Peprotech), and Recombinant Wnt5a (R&D systems) were used at 200 ng/ml, except when noted. Recombinant Dkk1 (R&D systems) was used at 200 ng/ml. JNK inhibitor SP600125 (Calbiochem, CAS# 129-56-6) was used at 10 µM, and iCRT3 (Calbiochem, pubchem # 126531502) [51] was used at 25 µM.

### Ethics Statement

This study was carried out in strict accordance with the recommendations in the Guide for the Care and Use of Laboratory Animals of the National Institutes of Health. The protocol was approved by the Institutional Animal Care and Use Committees of Weill Cornell Medical College (Protocol Number: 0052–11), and the New York Blood Center (Protocol Number: 267).

## Results

### MMTV-*Wnt1* transgenic mammospheres exhibit similar properties to those from wild-type mice

**Figure 1:**
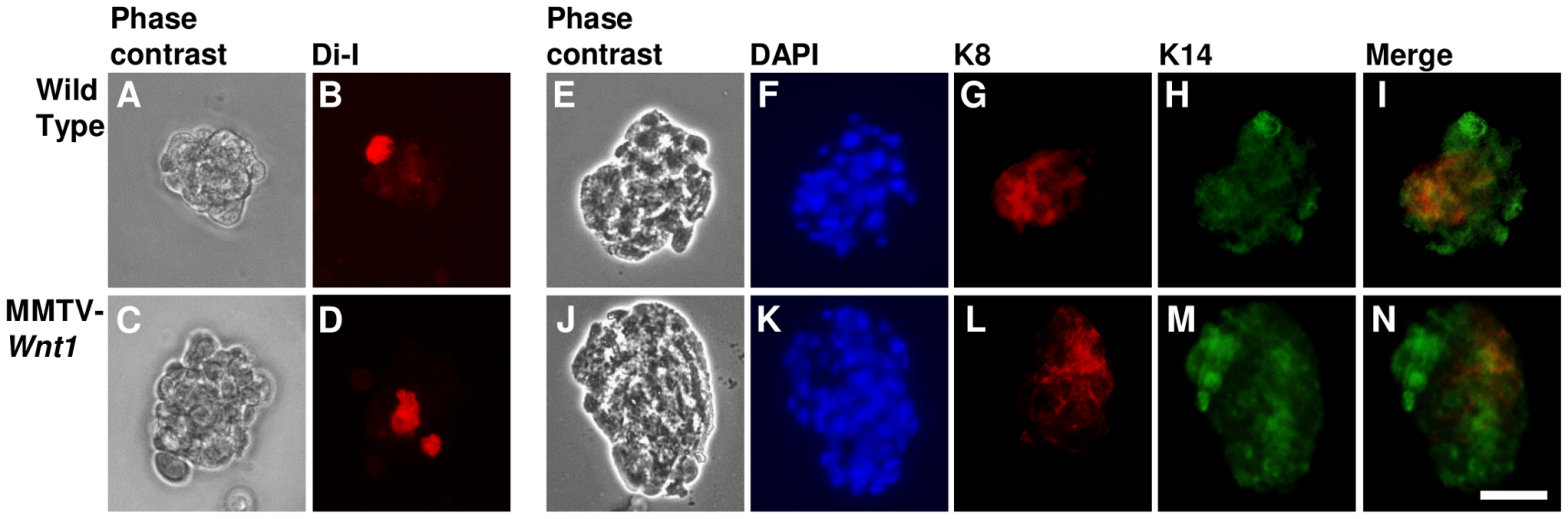
Wild-type and MMTV-*Wnt1* mammospheres contain newly replicated cells and have similar differentiation capacity. Phase contrast (A,C) and Di-I fluorescence images (B,D) of wild-type (A,B) and MMTV-*Wnt1* (C,D) mammospheres. Wild-type (E–I) and MMTV-*Wnt1* (J–N) mammospheres stained with DAPI (F, K), anti-cytokeratin K8 (luminal marker; G, L), anti-cytokeratin K14 (basal marker; H, M), and the merged image of K8 and K14 (I, N). Scale bar  = 50 µm.

To investigate the effects of Wnt-induced signaling in mouse mammary stem cells, we employed *ex vivo* mammosphere cultures derived from primary mouse mammary epithelium. Dissociated epithelial cells were obtained from wild-type mice and from MMTV-*Wnt1* transgenic animals. Such mammosphere cultures provide an assay system for stem cell-initiated sphere growth, independent of previously identified stem cell enrichment markers [12], [13]. Single cell suspensions being assayed for mammosphere formation were labeled with the lipid-soluble vital dye Di-I in order to track cell division. The majority of cells in all resultant secondary mammospheres exhibited low, or undetectable, fluorescence, the tracking dye having been diluted through multiple cell divisions (Figure 1A–D). In contrast, we observed bright fluorescence in one to two cells per mammosphere, suggesting that individual mammosphere forming cells can divide asymmetrically so as to retain the cell tracking dye in one daughter cell, while the majority of cells within each sphere are derived by serial proliferation. The lineage-specific markers Cytokeratin 8 (K8) and Cytokeratin 14 (K14) were used to identify luminal and basal mammary cells, respectively, in wild-type and MMTV-*Wnt1* derived mammospheres [12], [52]. For both genotypes, all mammospheres contained a mixture of cells expressing both K8 and K14, cells expressing K14 alone, and marker-negative cells. Most mammospheres also contained cells that expressed K8 alone (Figure 1E–N). This indicates that MMTV-*Wnt1* and wild-type mammospheres have similar capacity to produce progeny cells expressing differentiation markers during mammosphere growth *in vitro*. In addition, wild-type and MMTV-*Wnt1* mammospheres exhibited similar morphology in both shape and size (Figure 1). To confirm the expected self-renewal capacity of cells with mammosphere forming ability in these cultures, the number of mammosphere-forming units (MFUs) was measured at sequential passages (Figure S1). The continued capacity to form mammospheres was similar to that observed by others using comparable culture systems, indicating that the cultures contained cells with stem cell properties of differentiation and self-renewal [12], [53], [54]. In subsequent experiments we quantified the number of MFUs in secondary mammosphere assays, reflecting the number of cells with mammary stem cell properties [13], [55].

### Canonical Wnt Signaling promotes mammosphere formation

**Figure 2:**
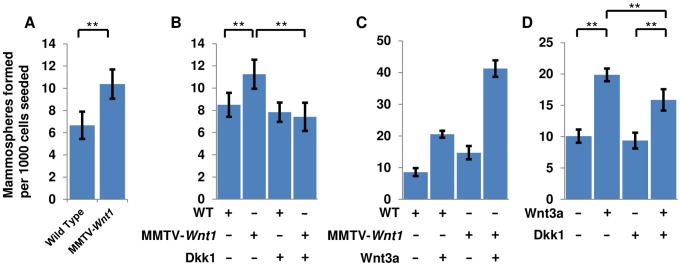
Canonical Wnt signaling increases the number of Mammosphere Forming Units. Secondary mammospheres were counted one week after plating dissociated cells treated as indicated. (A) Increased mammosphere formation in cultures derived from MMTV-*Wnt1* tissue versus wild-type. (B) Mammosphere numbers from wild-type and MMTV-*Wnt1* cultures with or without treatment with 200 ng/ml Dkk1. (C) Mammospheres from wild-type and MMTV-*Wnt1* cultures with or without treatment with 200 ng/ml Wnt3a. (D) Wild-type secondary mammospheres with or without recombinant Wnt3a, Dkk1, or both. **p<.01. All values in panel C were significantly different from one another at p<.01. Error bars show 95% confidence intervals.

In a variety of other stem cell assays systems, Wnt/β-catenin signaling has been associated with stem cell self-renewal or expansion [22], [56], [57]. Moreover, the lobuloalveolar mammary hyperplasia characteristic of MMTV-*Wnt1* transgenic mice has been reported to contain an increased absolute number of mammary stem cells, and an increased proportion of stem cells as defined by CD24^+^ CD29^HI^ surface markers [3]. To examine the consequences of canonical Wnt signaling in mammosphere assays, we measured the numbers of secondary MFUs in wild-type and MMTV-*Wnt1* cultures and observed a significantly larger number of MFUs per 1000 cells in MMTV-*Wnt1* cultures compared to wild type (Figure 2A). This indicates a greater percentage of cells with capacity for stem cell behavior in MMTV-*Wnt1* cultures compared to wild type.

The elevated number of MFUs in MMTV-*Wnt1 ex vivo* cultures might depend on a continuous Wnt1 signal, or Wnt1 signaling *in vivo* at an earlier stage might induce a change in cell fate such that mammosphere-forming capacity is permanently altered, irrespective of continued Wnt signaling. To distinguish between these possibilities, we treated dissociated primary mammosphere cells with Dkk1, a specific antagonist of Wnt binding to Lrp5/6, in order to block Wnt1 signaling [58]–[60]. The addition of Dkk1 to MMTV-*Wnt1* cultures reduced their sphere forming capacity to wild-type levels (Figure 2B). The effect of Wnt1 expression was thus reversible upon blockade of receptors for the canonical Wnt pathway, implying that a continued elevated level of Wnt/β-catenin signaling is required for the increase in MFU numbers observed in MMTV-*Wnt1* cultures.

We next tested whether acute stimulation of canonical Wnt signaling could substitute for the long term elevated canonical Wnt signaling resulting from the MMTV-*Wnt1* transgene. Disassociated mammospheres were therefore treated with a single dose of recombinant Wnt3a, a Wnt protein that is functionally interchangeable with Wnt1 in canonical signaling assays [35], [61]. A single application of Wnt protein was sufficient to induce a two-fold increase in MFUs in wild-type cultures, as assayed by secondary sphere formation (Figure 2C). We also found that addition of Wnt3a to MMTV-*Wnt1* cultures further increased the MFU numbers above the levels observed in MMTV-*Wnt1* cultures or wild-type cultures treated with Wnt3a. As expected, the elevation of MFU numbers induced by Wnt3a was impaired by pre-treatment of cells with Dkk1 (Figure 2D). Thus, short term stimulation with Wnt3a phenocopies the effects of an MMTV-*Wnt1* transgene in mammosphere cultures.

### Wnt5a-mediated signaling promotes mammosphere formation

**Figure 3:**
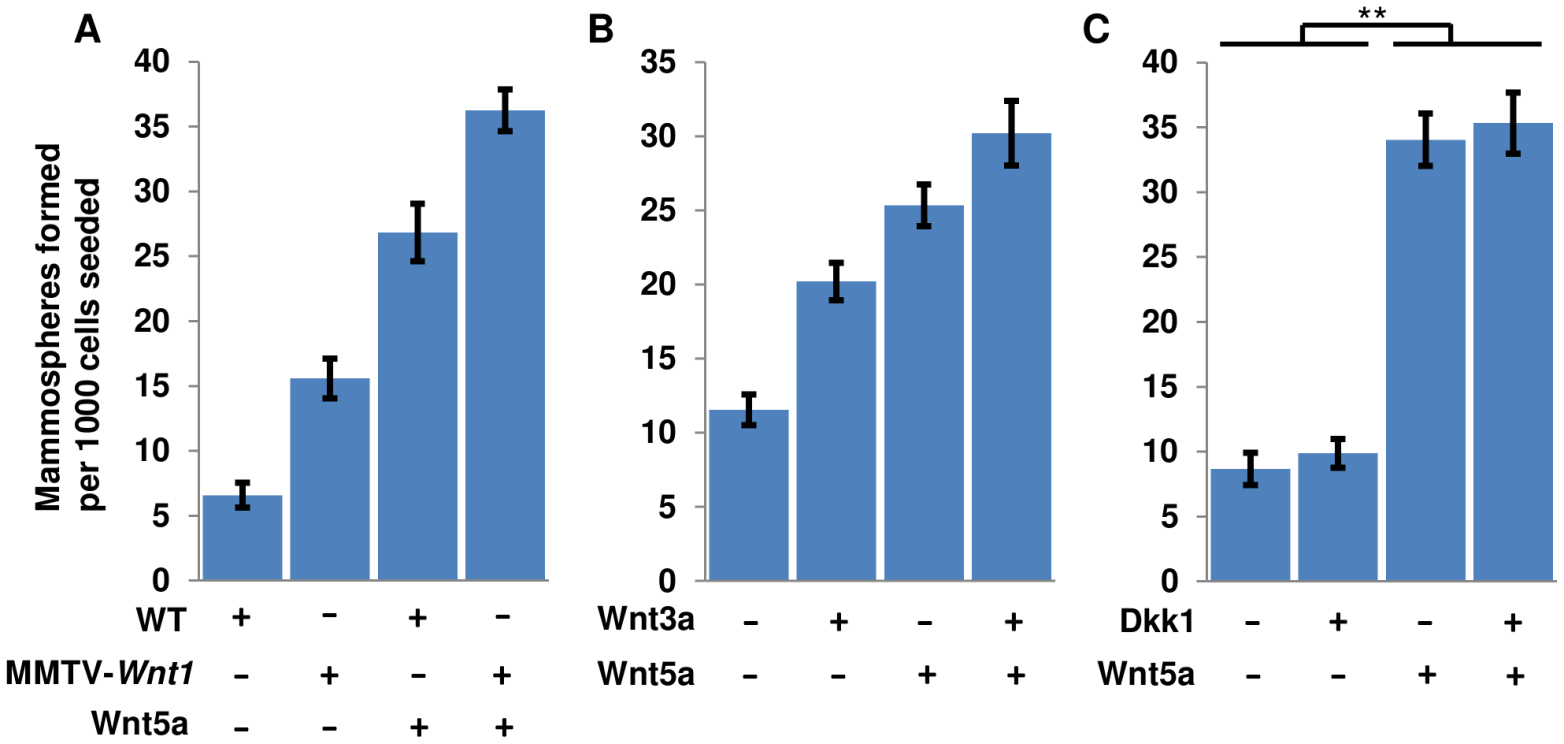
Wnt5a increases MFU number independently of canonical Wnt signaling. Dissociated cells were treated as indicated and secondary mammospheres were counted one week after plating. (A) Numbers of wild-type and MMTV-*Wnt1* secondary mammospheres after treatment with or without 200 ng/ml Wnt5a. (B) Wild-Type mammospheres treated with Wnt3a, Wnt5a or in combination. Wnt3a or Wnt5a were used individually at 400 ng/ml while in combination each was applied at 200 ng/ml. (C) Wild-type secondary mammosphere numbers after treatment with recombinant Wnt5a, Dkk1, or both (200 ng/ml each). In panels A and B all values were significantly different from one another at p<.01; in C, treatments with and without Wnt5a were significantly different at p<.01. Error bars show 95% confidence intervals.

In several cell systems, non-canonical signaling induced by Wnt5a can result in antagonism of canonical Wnt signaling [62]–[66]. Moreover, *in vivo* studies of Wnt5a signaling in the mammary gland suggest an antagonistic effect on ductal development [46], [47], [67], [68]. We therefore used recombinant Wnt5a to test the effects of non-canonical Wnt signaling on sphere formation, anticipating a negative effect. Surprisingly, we observed a dramatic increase in MFU number in response to Wnt5a treatment (Figure 3A). Moreover, when added to cultures from MMTV-*Wnt1* mice, recombinant Wnt5a further increased the number of MFUs beyond the elevated level induced by the MMTV-*Wnt1* transgene (Figure 3A). Similarly, when wild-type cultures were treated with recombinant Wnt3a and Wnt5a in combination, we observed an additive elevation of MFU numbers from the two ligands although each was applied at half the concentration used for each Wnt separately (Figure 3B). These results suggest that Wnt3a and Wnt5a independently promote mammosphere formation.

### Wnt5a-induced mammosphere formation results from non-canonical Wnt signaling

**Figure 4:**
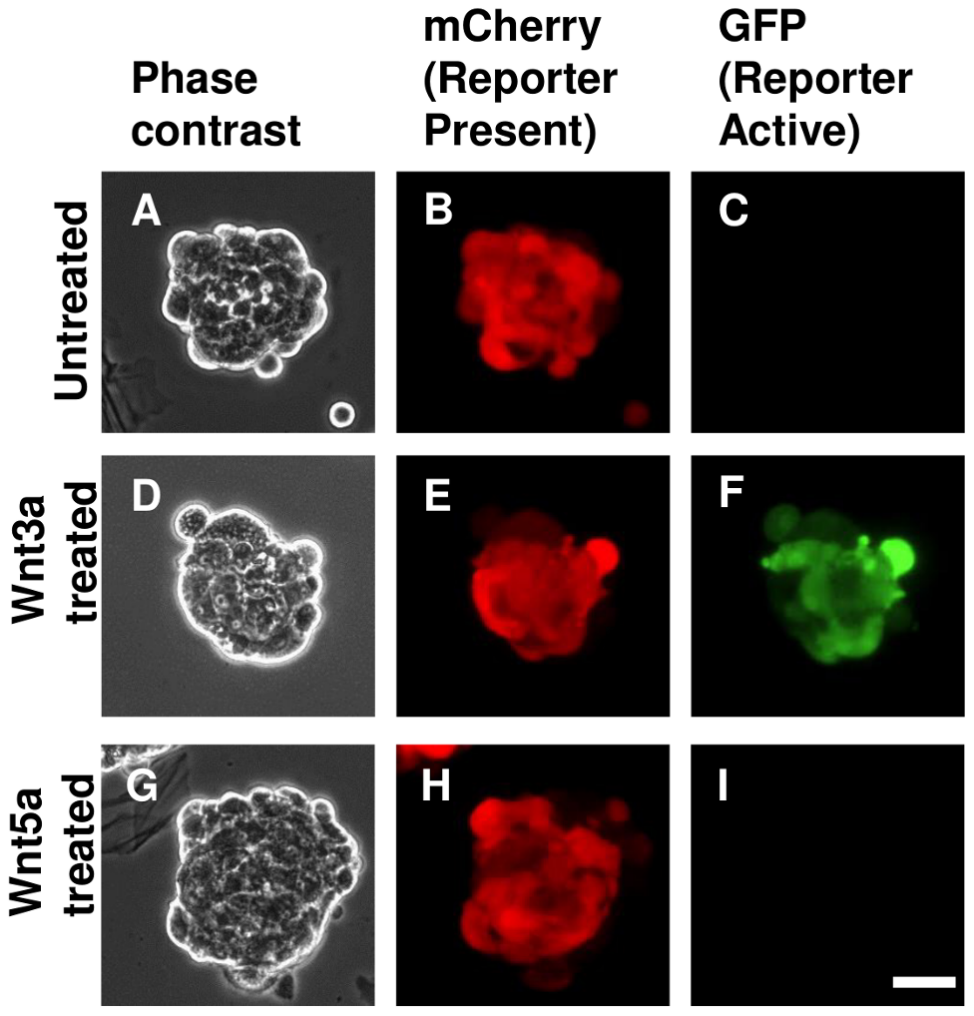
Wnt5a does not induce canonical Wnt signaling in mammospheres. Wild-type mammospheres were infected with the lentiviral reporter 7TGC and untreated (A–C), treated with 200 ng/ml Wnt3a (D–F), or treated with 200 ng/ml Wnt5a (G–I). Representative mammospheres imaged by phase contrast (A, D, and G), mCherry fluorescence (B, E, and H), and GFP fluorescence (C, F, and I). Constitutive mCherry expression indicates presence of the lentiviral reporter, while GFP fluorescence indicates activation of its β-catenin-TCF/LEF responsive promoter. Scale bar  = 50 µm. Numbers of spheres imaged: untreated, n = 86; Wnt3a treated, n = 79; Wnt5a treated, n = 103.

Signaling induced by Wnt5a typically acts via non-canonical pathways [43], [44]. However, under unusual circumstances, such as overexpression of Frizzled4 or 5, a canonical Wnt/β-catenin signal can be induced by Wnt5a [62], [69]. To test whether the MFU promoting effect of Wnt5a might be due to such signaling we blocked the canonical-specific Wnt receptors Lrp5 and Lrp6 by pretreatment of disassociated mammosphere cells with Dkk1 prior to stimulation with Wnt5a. This had no inhibitory effect on the response to Wnt5a, suggesting that Wnt5a does not act via Lrp5/6 in this assay (Figure 3C). To ensure that Wnt5a did not stimulate β-catenin/TCF activity independently of Lrp5/6, we infected secondary mammosphere cultures with a lentivirus (7TGC) that constitutively expresses mCherry, and expresses Green Fluorescent Protein (GFP) only in response to β-catenin-mediated transcriptional activation [49]. These cultures were then challenged with either recombinant Wnt3a or Wnt5a and mCherry-positive mammospheres were examined for GFP expression. While Wnt3a strongly induced the GFP reporter, no such induction was observed in Wnt5a-treated mammospheres or untreated controls (Figure 4). These results demonstrate that, unlike Wnt3a, Wnt5a failed to activate canonical Wnt/β-catenin signaling in mammospheres. We therefore conclude that Wnt5a mediates its effect through a non-canonical signaling pathway.

### Wnt5a stimulation of mammospheres requires *Ror2*

**Figure 5:**
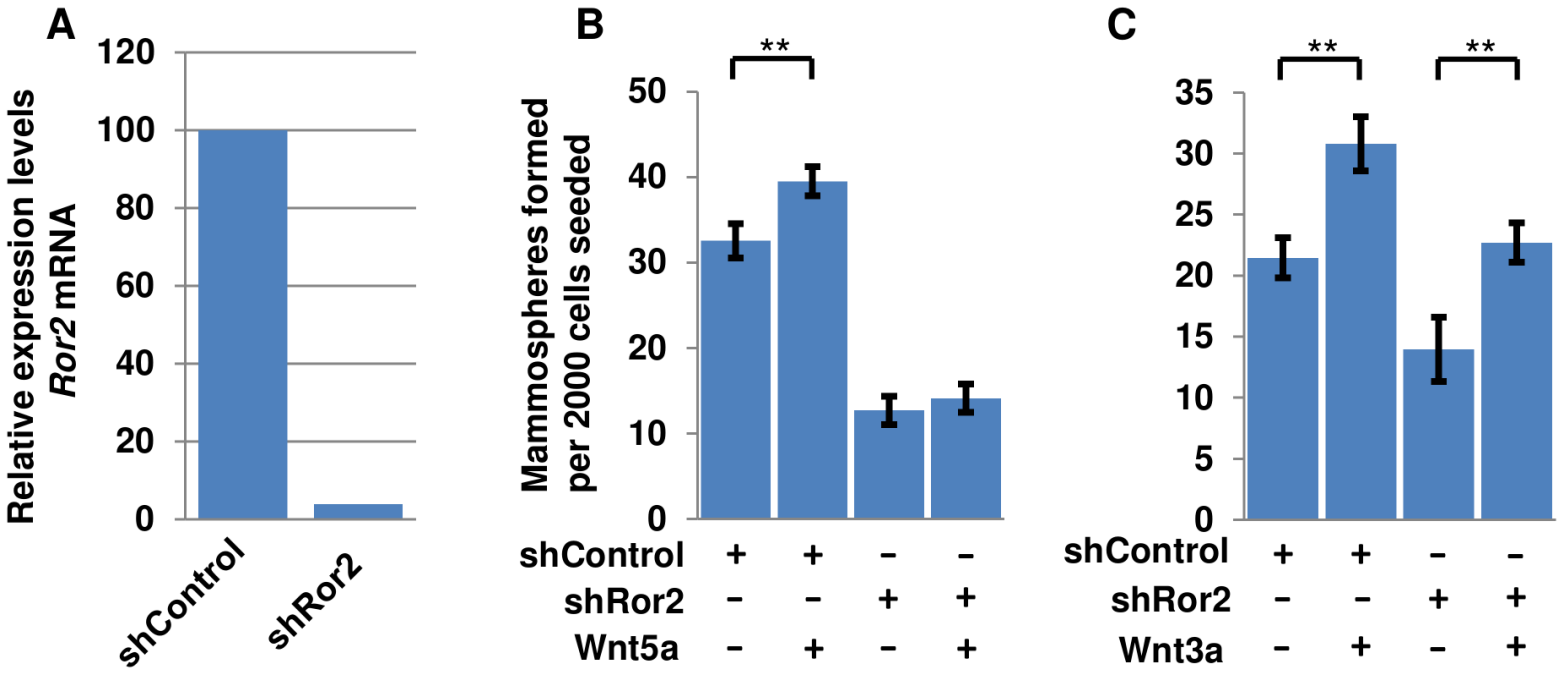
Knockdown of Ror2 by shRNA inhibits the increase in MFU numbers mediated by Wnt5a but not by Wnt3a. (A) Expression of *Ror2* mRNA measured by qPCR in mammospheres infected with control vector shControl or knockdown vector shRor2. (B) Wild-type mammosphere cells were infected with shRor2 or shControl lentiviral vectors and treated with or without 200 ng/ml Wnt5a. Secondary mammospheres were counted one week after plating. (C) Wild-type mammospheres infected with shRor2 or shControl lentiviral vector were treated with and without 200 ng/ml. **p<.01. Error bars show 95% confidence intervals.

One of the Wnt receptors specifically associated with non-canonical signaling is the tyrosine kinase Ror2, which has been shown to bind Wnt5a and to form a ternary complex with Frizzled proteins [42], [43], [70], [71]. To address whether the mammosphere-promoting function of Wnt5a is dependent on *Ror2*, we used a lentiviral *Ror2* shRNA construct (shRor2) to suppress *Ror2* expression, and validated its ability to knock down *Ror2* mRNA in mammospheres by qRT-PCR (Figure 5A) [50]. Secondary mammosphere cultures infected with shRor2 or a non-specific shRNA control vector were then treated with either Wnt5a or Wnt3a. In cultures infected with the control vector, Wnt3a and Wnt5a both increased the number of MFUs as in previous experiments. Infection with shRor2 reduced mammosphere formation compared to shControl. However, while cells infected with shRor2 responded to Wnt3a with increased numbers of MFUs, they failed to respond to Wnt5a in that no significant increase in MFUs was observed (Figure 5B, C). This indicates a requirement for *Ror2* in sphere formation mediated by Wnt5a but not by Wnt3a. It also suggests a basal function for *Ror2* in sphere formation in the absence of exogenous Wnts.

### Wnt5a signaling in mammospheres is dependent on JNK but not β-catenin/TCF

**Figure 6:**
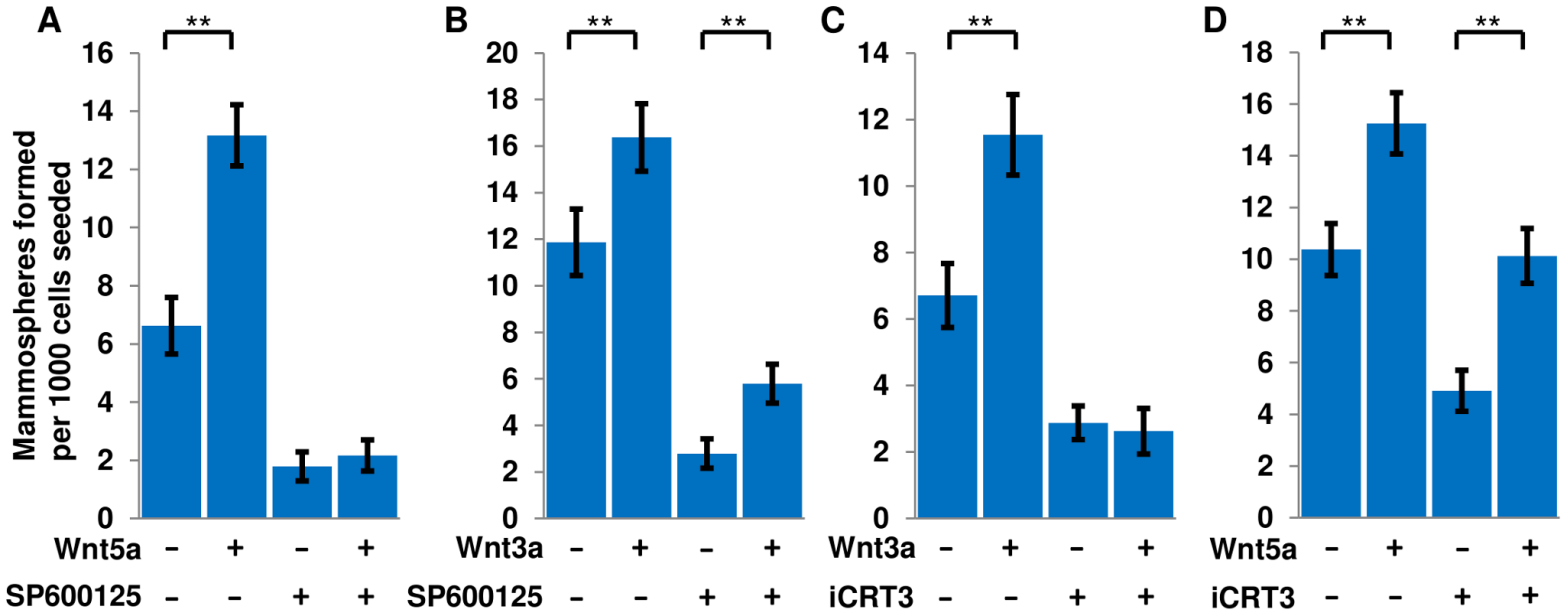
The Wnt5a-induced increase in MFU numbers is mediated via JNK, and not β-catenin/TCF. Secondary mammospheres were counted one week after plating wild-type cells with treatments indicated: (A) Wnt5a at 200 ng/ml, JNK inhibitor SP600125 at 10 µM, or both in combination; (B) Wnt3a at 200 ng/ml, SP600125 at 10 µM, or both in combination; (C) Wnt3a at 200 ng/ml, the β-catenin/TCF antagonist iCRT3 at 25 µM, or both in combination; (D) Wnt5a at 200 ng/ml, iCRT3 at 25 µM, or both in combination. **p<.01. Error bars show 95% confidence intervals.

Activation of JNK has been reported as an intracellular effector of non-canonical Wnt signaling, and has been specifically implicated downstream of Wnt5a and Ror2 [42], [72]. We therefore used a small molecule pan-JNK inhibitor, SP600125, to test whether activation of JNK is required for the effect of Wnt5a in mammosphere cultures [73], [74]. Addition of Wnt5a in the presence of JNK inhibitor failed to induce any increase in MFUs, while Wnt3a was still able to induce a two fold increase in MFU numbers in the presence of inhibitor (Figure 6A, B). In a complementary experiment we used iCRT3, a small molecule inhibitor of canonical Wnt signaling which specifically blocks the interaction between β-catenin and TCF/LEF proteins [51]. In secondary mammosphere assays incubated with iCRT3, Wnt3a failed to promote mammosphere formation, while Wnt5a increased the number of MFUs by two fold (Figure 6C, D). Both iCRT3 and JNK inhibitor treatment inhibited mammosphere formation irrespective of exogenous ligands. These results demonstrate a requirement for JNK activity in mediating the effects of Wnt5a, but not Wnt3a, on mammosphere formation. Conversely, they suggest that the increase in MFU numbers mediated by Wnt3a requires the interaction of β-catenin and TCF/LEF, while Wnt5a acts independently of β-catenin-mediated transcription. Additionally mammosphere formation may be dependent upon a basal level of both canonical and non-canonical Wnt signaling. Together, these results indicate that Wnt5a and Wnt3a promote mammosphere formation through distinct signaling mechanisms.

## Discussion

In this study we have examined the effects of Wnt signals on secondary mammosphere formation, an *in vitro* assay that reflects mammary stem cell activity. Using *ex vivo* cultures from both wild-type and MMTV-*Wnt1* transgenic mice, we observed that canonical Wnt/β-catenin signaling stimulates mammosphere formation by primary mouse mammary epithelial cells. This is evident from comparing mammosphere formation by MMTV-*Wnt1* and wild-type *ex vivo* cultures, and from the effect of recombinant Wnt3a on wild-type cells. We also observed that treatment with Wnt5a caused a similar increase in mammosphere formation, although it did not stimulate the Wnt/β-catenin pathway. Instead, the effects of Wnt5a were mediated via a non-canonical Wnt signaling pathway acting via the receptor Ror2 and dependent on activity of the kinase JNK. Our results indicate that both canonical and non-canonical Wnt signals act independently to promote the stem cell properties required for mammosphere formation.

Previous studies of the effects of canonical Wnt signaling on mouse mammary epithelial stem cells, both *in vivo* and *in vitro*, have focused on the sub-population of cells with the immunophenotype CD24^+^ CD29^hi^, which are enriched for cells capable of mammary gland repopulation [3], [35], [75]. Wnt3a promotes the self-renewal of such cells *in vitro* and their abundance *in vivo* is elevated in MMTV-*Wnt1* transgenic mice [3], [35]. The present studies constitute a complementary approach in focusing on the mammosphere-forming capacity of mammary stem cells independently of specific cell surface markers. We found that a single dose of Wnt3a protein applied to dissociated cells was sufficient to increase secondary mammosphere formation. Consistent with this, and with the results of Shackleton *et al*. [3], mammary epithelium from MMTV-*Wnt1* mice displayed a greater number of MFUs *in vitro* than equivalent cultures from wild-type mice. This effect was blocked by addition of Dkk1 to the *Wnt1* transgenic cultures. This indicates that elevated canonical Wnt signaling is actively required during *ex vivo* culture in order to produce increased numbers of MFUs, rather than it arising from a permanent change in cell fate mediated by the *Wnt1* transgene during early mammary development. The immediate mammary phenotype of MMTV-*Wnt1* mice is precocious and permanent lobuloalveolar hyperplasia, which imparts significant risk of progression to carcinoma [34]. If the hyperplasia is a consequence of increased numbers of stem-like cells, our data suggest that this would be reversible upon blockade of canonical Wnt signaling. Moreover a continuing effect of canonical Wnt signaling acting on tumor stem cells might account for the suppression of tumor growth in MMTV-*Wnt1* transgenic mice when the Wnt1 signal is antagonized after progression to carcinoma [76]–[78].

While elevated levels of canonical Wnt/β-catenin signaling are clearly associated with promoting hyperplasia and tumorigenesis in the mammary gland, both in mice and humans [20], [23], [25], the effects of non-canonical Wnt signaling, e.g. as elicited by Wnt5a, have generally been linked to inhibitory effects. In human breast cancer, for example, loss of Wnt5a expression correlates with poor prognosis, suggesting that the gene acts as a tumor suppressor [79], [80]. Moreover, Wnt5a protein can inhibit ductal proliferation in the mouse mammary gland, loss of *Wnt5a* confers a more aggressive mammary tumor phenotype, and there is evidence that Wnt5a can antagonize the intracellular pathway of canonical Wnt signaling in several settings [25], [46], [47], [62], [65]. Given this background, we were surprised to observe a significant increase in MFU numbers upon treatment with Wnt5a in mammosphere assays, an effect comparable to that of Wnt1 or Wnt3a which each stimulate the canonical Wnt pathway [38], [62]. Nevertheless our results are consistent with those of Scheel *et al*. (2011), who observed that treatment of human breast epithelial cells with Wnt5a, in conjunction with activation of Wnt/β-catenin and TGFβ pathways, enhanced the efficiency of mammosphere formation as well inducing the expression of EMT markers [81]. In addition, it has been shown that Wnt5a promotes the self-renewal of spermatogonial stem cells *in vitro*, suggesting that its positive effect on mammospheres is not unique to breast tissues [82]. Given the numerous distinct signaling pathways and receptors through which Wnt5a may act [39], [40], [83], the positive effects on mammosphere formation observed in our study can perhaps be reconciled with the growth suppressive effects of Wnt5a signaling in mammary tissue [46], [47] by invoking distinct signaling responses to Wnt5a in stem cells versus committed progenitors.

Although Wnt5a typically signals via β-catenin-independent mechanisms, there are special circumstances in which it has been found to activate the canonical Wnt/β-catenin pathway [62], [69], [84]. This was not the case in our experiments, however, since Wnt5a treatment of mammospheres failed to activate a transcriptional reporter of Wnt/β-catenin signaling, and the positive effect of Wnt5a was not blocked by either the Lrp5/6 antagonist Dkk1 or the β-Catenin/TCF inhibitor iCRT3. Instead, we provide evidence that stimulation of mammosphere formation by Wnt5a depends on *Ror2*, a receptor tyrosine kinase that binds Wnt5a and transduces a non-canonical Wnt signal that includes activation of JNK [42], [62], [72], [83]. Consistent with *Ror2* involvement, we found that Wnt5a-induced mammosphere formation was abolished by inhibition of JNK. We conclude that while Wnt3a promotes mammosphere formation via canonical Wnt signaling, Wnt5a does so by a non-canonical mechanism. Moreover, our data particularly implicate a *Ror2*-JNK pathway among the numerous other pathways that have been ascribed to non-canonical Wnt signaling [39], [83], [84].

It remains to be determined whether the independent and additive effects of canonical and non-canonical Wnt signaling on mammosphere formation are caused by both signals operating on the same population of mammosphere-forming cells, or whether they act on distinct target populations. Recent studies aimed at identifying mouse mammary stem cells by lineage tracing *in vivo* have yielded data that may be inconsistent with those from classical mammary reconstitution assays [5]–[7], [85]. Collectively these reports suggest that the capacity to act as multipotent stem cells may reside in several distinct cell types in the developing mammary gland and that they may be activated under different circumstances in response to hormonal signals, pregnancy, tissue damage, or other forms of stress. Thus, the stem cell phenotype may be subject to considerable plasticity in response to extrinsic factors such as Wnts and other putative stem cell niche components [86], [87]. Against the growing complexity of mammary stem cell analysis *in vivo*, mammosphere assays provide a promising system for dissecting the responses of individual self-renewing cells to defined factors *in vitro*.

## Supporting Information

Figure S1 **Wild-type mammospheres can be serially passaged for multiple generations.** Wild-type mammosphere cultures were serially passaged weekly. The number of mammospheres formed per 2000 cells plated was assayed at each passage. Passage one represents the number of secondary mammospheres resulting from passage from primary to secondary culture. Error bars show 95% confidence intervals.
(TIF)

## References

[1] Daniel CW, Ome KBD, Young JT, Blair PB, Faulkin LJ (1968) The in vivo life span of normal and preneoplastic mouse mammary glands: a serial transplantation study. PNAS 61: 53–60. 4301594 10.1073/pnas.61.1.53 PMC285904

[2] Kordon EC, Smith GH (1998) An entire functional mammary gland may comprise the progeny from a single cell. Development 125: 1921–1930. 9550724 10.1242/dev.125.10.1921

[3] Shackleton M, Vaillant F, Simpson KJ, Stingl J, Smyth GK, et al (2006) Generation of a functional mammary gland from a single stem cell. Nature 439: 84–88. 16397499 10.1038/nature04372

[4] Stingl J, Eirew P, Ricketson I, Shackleton M, Vaillant F, et al (2006) Purification and unique properties of mammary epithelial stem cells. Nature 439: 993–997. 16395311 10.1038/nature04496

[5] De Visser KE, Ciampricotti M, Michalak E, Wei-Min Tan D, Speksnijder EN, et al (2012) Developmental stage-specific contribution of LGR5+ cells to basal and luminal epithelial lineages in the postnatal mammary gland. The Journal of Pathology 228: 300–309. 22926799 10.1002/path.4096

[6] Van Keymeulen A, Rocha AS, Ousset M, Beck B, Bouvencourt G, et al (2011) Distinct stem cells contribute to mammary gland development and maintenance. Nature 479: 189–193. 21983963 10.1038/nature10573

[7] Rios AC, Fu NY, Lindeman GJ, Visvader JE (2014) In situ identification of bipotent stem cells in the mammary gland. Nature 506: 322–327. 24463516 10.1038/nature12948

[8] Méndez-Ferrer S, Michurina TV, Ferraro F, Mazloom AR, Macarthur BD, et al (2010) Mesenchymal and haematopoietic stem cells form a unique bone marrow niche. Nature 466: 829–834. 20703299 10.1038/nature09262 PMC3146551

[9] Shahi P, Seethammagari MR, Valdez JM, Xin L, Spencer DM (2011) Wnt and Notch pathways have interrelated opposing roles on prostate progenitor cell proliferation and differentiation. Stem Cells 29: 678–688. 21308863 10.1002/stem.606 PMC3148789

[10] Jensen JB, Parmar M (2006) Strengths and limitations of the neurosphere culture system. Mol Neurobiol 34: 153–161. 17308349 10.1385/MN:34:3:153

[11] Ahmed S (2009) The culture of neural stem cells. Journal of Cellular Biochemistry 106: 1–6. 19021147 10.1002/jcb.21972

[12] Dontu G, Abdallah WM, Foley JM, Jackson KW, Clarke MF, et al (2003) In vitro propagation and transcriptional profiling of human mammary stem/progenitor cells. Genes Dev 17: 1253–1270. 12756227 10.1101/gad.1061803 PMC196056

[13] Liao MJ, Zhang CC, Zhou B, Zimonjic DB, Mani SA, et al (2007) Enrichment of a population of mammary gland cells that form mammospheres and have in vivo repopulating activity. Cancer Res 67: 8131–8138. 17804725 10.1158/0008-5472.CAN-06-4493

[14] Liu S, Dontu G, Mantle ID, Patel S, Ahn N, et al (2006) Hedgehog signaling and Bmi-1 regulate self-renewal of normal and malignant human mammary stem cells. Cancer Res 66: 6063–6071. 16778178 10.1158/0008-5472.CAN-06-0054 PMC4386278

[15] Zhang M, Behbod F, Atkinson RL, Landis MD, Kittrell F, et al (2008) Identification of tumor-initiating cells in a p53-null mouse model of breast cancer. Cancer Res 68: 4674–4682. 18559513 10.1158/0008-5472.CAN-07-6353 PMC2459340

[16] Korkaya H, Paulson A, Iovino F, Wicha MS (2008) HER2 regulates the mammary stem/progenitor cell population driving tumorigenesis and invasion. Oncogene 27: 6120–6130. 18591932 10.1038/onc.2008.207 PMC2602947

[17] Dong Q, Wang D, Bandyopadhyay A, Gao H, Gorena KM, et al (2013) Mammospheres from murine mammary stem cell-enriched basal cells: Clonal characteristics and repopulating potential. Stem Cell Res 10: 396–404. 23466563 10.1016/j.scr.2013.01.007 PMC3622180

[18] Grimshaw MJ, Cooper L, Papazisis K, Coleman JA, Bohnenkamp HR, et al (2008) Mammosphere culture of metastatic breast cancer cells enriches for tumorigenic breast cancer cells. Breast Cancer Res 10: R52. 18541018 10.1186/bcr2106 PMC2481500

[19] Lamb R, Ablett MP, Spence K, Landberg G, Sims AH, et al (2013) Wnt pathway activity in breast cancer sub-types and stem-like cells. PLoS ONE 8: e67811. 23861811 10.1371/journal.pone.0067811 PMC3701602

[20] Brennan KR, Brown AMC (2004) Wnt proteins in mammary development and cancer. J Mammary Gland Biol Neoplasia 9: 119–131. 15300008 10.1023/B:JOMG.0000037157.94207.33

[21] Many AM, Brown AMC (2010) Mammary stem cells and cancer: roles of Wnt signaling in plain view. Breast Cancer Res 12: 313. 20887643 10.1186/bcr2631 PMC3096951

[22] Clevers H, Nusse R (2012) Wnt/β-Catenin Signaling and Disease. Cell 149: 1192–1205. 22682243 10.1016/j.cell.2012.05.012

[23] Howe LR, Brown AMC (2004) Wnt signaling and breast cancer. Cancer Biol Ther 3: 36–41. 14739782 10.4161/cbt.3.1.561

[24] Nusse R, Fuerer C, Ching W, Harnish K, Logan C, et al (2008) Wnt signaling and stem cell control. Cold Spring Harb Symp Quant Biol 73: 59–66. 19028988 10.1101/sqb.2008.73.035

[25] Van Camp JK, Beckers S, Zegers D, Van Hul W (2013) Wnt Signaling and the Control of Human Stem Cell Fate. Stem Cell Rev 10: 207–229. 10.1007/s12015-013-9486-8 24323281

[26] Polakis P (2007) The many ways of Wnt in cancer. Curr Opin Genet Dev 17: 45–51. 17208432 10.1016/j.gde.2006.12.007

[27] Li Y, Hively WP, Varmus HE (2000) Use of MMTV-Wnt-1 transgenic mice for studying the genetic basis of breast cancer. Oncogene 19: 1002–1009. 10713683 10.1038/sj.onc.1203273

[28] Li Y, Welm B, Podsypanina K, Huang S, Chamorro M, et al (2003) Evidence that transgenes encoding components of the Wnt signaling pathway preferentially induce mammary cancers from progenitor cells. Proc Natl Acad Sci U S A 100: 15853–15858. 14668450 10.1073/pnas.2136825100 PMC307657

[29] Liu BY, McDermott SP, Khwaja SS, Alexander CM (2004) The transforming activity of Wnt effectors correlates with their ability to induce the accumulation of mammary progenitor cells. Proc Natl Acad Sci U S A 101: 4158–4163. 15020770 10.1073/pnas.0400699101 PMC384711

[30] Incassati A, Chandramouli A, Eelkema R, Cowin P (2010) Key signaling nodes in mammary gland development and cancer: β-catenin. Breast Cancer Res 12: 213. 21067528 10.1186/bcr2723 PMC3046427

[31] Baker R, Kent CV, Silbermann RA, Hassell JA, Young LJT, et al (2010) Pea3 transcription factors and wnt1-induced mouse mammary neoplasia. PLoS ONE 5: e8854. 20107508 10.1371/journal.pone.0008854 PMC2809747

[32] Teissedre B, Pinderhughes A, Incassati A, Hatsell SJ, Hiremath M, et al (2009) MMTV-Wnt1 and ΔN89β-catenin induce canonical signaling in distinct progenitors and differentially activate Hedgehog signaling within mammary tumors. PLoS ONE 4: e4537. 19225568 10.1371/journal.pone.0004537 PMC2639708

[33] Badders NM, Goel S, Clark RJ, Klos KS, Kim S, et al (2009) The Wnt receptor, Lrp5, is expressed by mouse mammary stem cells and is required to maintain the basal lineage. PLoS ONE 4: e6594. 19672307 10.1371/journal.pone.0006594 PMC2720450

[34] Tsukamoto AS, Grosschedl R, Guzman RC, Parslow T, Varmus HE (1988) Expression of the int-1 gene in transgenic mice is associated with mammary gland hyperplasia and adenocarcinomas in male and female mice. Cell 55: 619–625. 3180222 10.1016/0092-8674(88)90220-6

[35] Zeng YA, Nusse R (2010) Wnt proteins are self-renewal factors for mammary stem cells and promote their long-term expansion in culture. Cell Stem Cell 6: 568–577. 20569694 10.1016/j.stem.2010.03.020 PMC2917779

[36] Van Amerongen R, Bowman AN, Nusse R (2012) Developmental stage and time dictate the fate of Wnt/β-catenin-responsive stem cells in the mammary gland. Cell Stem Cell 11: 387–400. 22863533 10.1016/j.stem.2012.05.023 PMC13155203

[37] Macdonald BT, Semenov MV, He X (2007) SnapShot: Wnt/β-catenin signaling. Cell 131: 1204. 18083108 10.1016/j.cell.2007.11.036

[38] Shimizu H, Julius MA, Giarre M, Zheng Z, Brown AMC, et al (1997) Transformation by Wnt family proteins correlates with regulation of β-catenin. Cell Growth Differ 8: 1349–1358. 9419423

[39] Semenov MV, Habas R, Macdonald BT, He X (2007) SnapShot: Noncanonical Wnt Signaling Pathways. Cell 131: 1378. 18160045 10.1016/j.cell.2007.12.011

[40] Van Amerongen R (2012) Alternative Wnt pathways and receptors. Cold Spring Harb Perspect Biol 4: a007914. 22935904 10.1101/cshperspect.a007914 PMC3475174

[41] Liu G, Bafico A, Aaronson SA (2005) The mechanism of endogenous receptor activation functionally distinguishes prototype canonical and noncanonical Wnts. Mol Cell Biol 25: 3475–3482. 15831454 10.1128/MCB.25.9.3475-3482.2005 PMC1084300

[42] Oishi I, Suzuki H, Onishi N, Takada R, Kani S, et al (2003) The receptor tyrosine kinase Ror2 is involved in non-canonical Wnt5a/JNK signalling pathway. Genes Cells 8: 645–654. 12839624 10.1046/j.1365-2443.2003.00662.x

[43] Grumolato L, Liu G, Mong P, Mudbhary R, Biswas R, et al (2010) Canonical and noncanonical Wnts use a common mechanism to activate completely unrelated coreceptors. Genes Dev 24: 2517–2530. 21078818 10.1101/gad.1957710 PMC2975928

[44] Gonzalez-Sancho JM, Brennan KR, Castelo-Soccio LA, Brown AMC (2004) Wnt proteins induce dishevelled phosphorylation via an LRP5/6- independent mechanism, irrespective of their ability to stabilize β-catenin. Mol Cell Biol 24: 4757–4768. 15143170 10.1128/MCB.24.11.4757-4768.2004 PMC416421

[45] McDonald SL, Silver A (2009) The opposing roles of Wnt-5a in cancer. Br J Cancer 101: 209–214. 19603030 10.1038/sj.bjc.6605174 PMC2720208

[46] Roarty K, Serra R (2007) Wnt5a is required for proper mammary gland development and TGFβ-mediated inhibition of ductal growth. Development 134: 3929–3939. 17898001 10.1242/dev.008250

[47] Roarty K, Baxley SE, Crowley MR, Frost AR, Serra R (2009) Loss of TGF-β or Wnt5a results in an increase in Wnt/β-catenin activity and redirects mammary tumour phenotype. Breast Cancer Res 11: R19. 19344510 10.1186/bcr2244 PMC2688948

[48] Dontu G, Jackson KW, McNicholas E, Kawamura MJ, Abdallah WM, et al (2004) Role of Notch signaling in cell-fate determination of human mammary stem/progenitor cells. Breast Cancer Research 6: R605. 15535842 10.1186/bcr920 PMC1064073

[49] Fuerer C, Nusse R (2010) Lentiviral Vectors to Probe and Manipulate the Wnt Signaling Pathway. PLoS ONE 5: e9370. 20186325 10.1371/journal.pone.0009370 PMC2826402

[50] Miyoshi H, Ajima R, Luo CT, Yamaguchi TP, Stappenbeck TS (2012) Wnt5a potentiates TGF-β signaling to promote colonic crypt regeneration after tissue injury. Science 338: 108–113. 22956684 10.1126/science.1223821 PMC3706630

[51] Gonsalves FC, Klein K, Carson BB, Katz S, Ekas LA, et al (2011) An RNAi-based chemical genetic screen identifies three small-molecule inhibitors of the Wnt/wingless signaling pathway. Proceedings of the National Academy of Sciences 108: 5954–5963. 10.1073/pnas.1017496108 PMC3076864 21393571

[52] Mani SA, Guo W, Liao MJ, Eaton EN, Ayyanan A, et al (2008) The epithelial-mesenchymal transition generates cells with properties of stem cells. Cell 133: 704–715. 18485877 10.1016/j.cell.2008.03.027 PMC2728032

[53] Dey D, Saxena M, Paranjape AN, Krishnan V, Giraddi R, et al (2009) Phenotypic and functional characterization of human mammary stem/progenitor cells in long term culture. PLoS One 4: e5329. 19390630 10.1371/journal.pone.0005329 PMC2669709

[54] Tao L, Roberts AL, Dunphy KA, Bigelow C, Yan H, et al (2011) Repression of Mammary Stem/Progenitor Cells by p53 Is Mediated by Notch and Separable from Apoptotic Activity. Stem Cells 29: 119–127. 21280161 10.1002/stem.552 PMC3404152

[55] Moraes RC, Zhang X, Harrington N, Fung JY, Wu M-F, et al (2007) Constitutive activation of smoothened (SMO) in mammary glands of transgenic mice leads to increased proliferation, altered differentiation and ductal dysplasia. Development 134: 1231–1242. 17287253 10.1242/dev.02797

[56] Ling L, Nurcombe V, Cool SM (2009) Wnt signaling controls the fate of mesenchymal stem cells. Gene 433: 1–7. 19135507 10.1016/j.gene.2008.12.008

[57] Holland JD, Klaus A, Garratt AN, Birchmeier W (2013) Wnt signaling in stem and cancer stem cells. Current Opinion in Cell Biology 25: 254–264. 23347562 10.1016/j.ceb.2013.01.004

[58] Semënov MV, Tamai K, Brott BK, Kühl M, Sokol S, et al (2001) Head inducer Dickkopf-1 is a ligand for Wnt coreceptor LRP6. Curr Biol 11: 951–961. 11448771 10.1016/s0960-9822(01)00290-1

[59] Bafico A, Liu G, Yaniv A, Gazit A, Aaronson SA (2001) Novel mechanism of Wnt signalling inhibition mediated by Dickkopf-1 interaction with LRP6/Arrow. Nat Cell Biol 3: 683–686. 11433302 10.1038/35083081

[60] Mao B, Wu W, Li Y, Hoppe D, Stannek P, et al (2001) LDL-receptor-related protein 6 is a receptor for Dickkopf proteins. Nature 411: 321–325. 11357136 10.1038/35077108

[61] Baljinnyam B, Klauzinska M, Saffo S, Callahan R, Rubin JS (2012) Recombinant R-spondin2 and Wnt3a up- and down-regulate novel target genes in C57MG mouse mammary epithelial cells. PLoS ONE 7: e29455. 22238613 10.1371/journal.pone.0029455 PMC3251591

[62] Mikels AJ, Nusse R (2006) Purified Wnt5a protein activates or inhibits β-catenin-TCF signaling depending on receptor context. PLoS Biol 4: e115. 16602827 10.1371/journal.pbio.0040115 PMC1420652

[63] Nemeth MJ, Topol L, Anderson SM, Yang Y, Bodine DM (2007) Wnt5a inhibits canonical Wnt signaling in hematopoietic stem cells and enhances repopulation. Proc Natl Acad Sci USA 104: 15436–15441. 17881570 10.1073/pnas.0704747104 PMC1986571

[64] Van Amerongen R, Fuerer C, Mizutani M, Nusse R (2012) Wnt5a can both activate and repress Wnt/β-catenin signaling during mouse embryonic development. Dev Biol 369: 101–114. 22771246 10.1016/j.ydbio.2012.06.020 PMC3435145

[65] Topol L, Jiang X, Choi H, Garrett-Beal L, Carolan PJ, et al (2003) Wnt-5a inhibits the canonical Wnt pathway by promoting GSK-3-independent β-catenin degradation. J Cell Biol 162: 899–908. 12952940 10.1083/jcb.200303158 PMC2172823

[66] Baksh D, Boland GM, Tuan RS (2007) Cross-talk between Wnt signaling pathways in human mesenchymal stem cells leads to functional antagonism during osteogenic differentiation. J Cell Biochem 101: 1109–1124. 17546602 10.1002/jcb.21097

[67] Serra R, Easter SL, Jiang W, Baxley SE (2011) Wnt5a as an effector of TGFβ in mammary development and cancer. J Mammary Gland Biol Neoplasia 16: 157–167. 21416313 10.1007/s10911-011-9205-5 PMC3107509

[68] Pavlovich AL, Boghaert E, Nelson CM (2011) Mammary branch initiation and extension are inhibited by separate pathways downstream of TGFβ in culture. Exp Cell Res 317: 1872–1884. 21459084 10.1016/j.yexcr.2011.03.017 PMC3123406

[69] He X, Saint-Jeannet JP, Wang Y, Nathans J, Dawid I, et al (1997) A member of the Frizzled protein family mediating axis induction by Wnt-5A. Science 275: 1652–1654. 9054360 10.1126/science.275.5306.1652

[70] Nishita M, Itsukushima S, Nomachi A, Endo M, Wang Z, et al (2010) Ror2/Frizzled complex mediates Wnt5a-induced AP-1 activation by regulating Dishevelled polymerization. Mol Cell Biol 30: 3610–3619. 20457807 10.1128/MCB.00177-10 PMC2897551

[71] O'Connell MP, Fiori JL, Xu M, Carter AD, Frank BP, et al (2010) The orphan tyrosine kinase receptor, ROR2, mediates Wnt5A signaling in metastatic melanoma. Oncogene 29: 34–44. 19802008 10.1038/onc.2009.305 PMC2803338

[72] Nomachi A, Nishita M, Inaba D, Enomoto M, Hamasaki M, et al (2008) Receptor tyrosine kinase Ror2 mediates Wnt5a-induced polarized cell migration by activating c-Jun N-terminal kinase via actin-binding protein filamin A. J Biol Chem 283: 27973–27981. 18667433 10.1074/jbc.M802325200

[73] Bennett BL, Sasaki DT, Murray BW, O'Leary EC, Sakata ST, et al (2001) SP600125, an anthrapyrazolone inhibitor of Jun N-terminal kinase. Proc Natl Acad Sci USA 98: 13681–13686. 11717429 10.1073/pnas.251194298 PMC61101

[74] Han Z, Boyle DL, Chang L, Bennett B, Karin M, et al (2001) c-Jun N-terminal kinase is required for metalloproteinase expression and joint destruction in inflammatory arthritis. J Clin Invest 108: 73–81. 11435459 10.1172/JCI12466 PMC209341

[75] Cho RW, Wang X, Diehn M, Shedden K, Chen GY, et al (2008) Isolation and molecular characterization of cancer stem cells in MMTV-Wnt-1 murine breast tumors. Stem Cells 26: 364–371. 17975224 10.1634/stemcells.2007-0440

[76] Ettenberg SA, Charlat O, Daley MP, Liu S, Vincent KJ, et al (2010) Inhibition of tumorigenesis driven by different Wnt proteins requires blockade of distinct ligand-binding regions by LRP6 antibodies. Proc Natl Acad Sci USA 107: 15473–15478. 20713706 10.1073/pnas.1007428107 PMC2932603

[77] DeAlmeida VI, Miao L, Ernst JA, Koeppen H, Polakis P, et al (2007) The soluble wnt receptor Frizzled8CRD-hFc inhibits the growth of teratocarcinomas in vivo. Cancer Res 67: 5371–5379. 17545618 10.1158/0008-5472.CAN-07-0266

[78] Gunther EJ, Moody SE, Belka GK, Hahn KT, Innocent N, et al (2003) Impact of p53 loss on reversal and recurrence of conditional Wnt-induced tumorigenesis. Genes Dev 17: 488–501. 12600942 10.1101/gad.1051603 PMC195997

[79] Jonsson M, Dejmek J, Bendahl PO, Andersson T (2002) Loss of Wnt-5a protein is associated with early relapse in invasive ductal breast carcinomas. Cancer Res 62: 409–416. 11809689

[80] Dejmek J, Dejmek A, Säfholm A, Sjölander A, Andersson T (2005) Wnt-5a protein expression in primary dukes B colon cancers identifies a subgroup of patients with good prognosis. Cancer Res 65: 9142–9146. 16230369 10.1158/0008-5472.CAN-05-1710

[81] Scheel C, Eaton EN, Li SH-J, Chaffer CL, Reinhardt F, et al (2011) Paracrine and autocrine signals induce and maintain mesenchymal and stem cell states in the breast. Cell 145: 926–940. 21663795 10.1016/j.cell.2011.04.029 PMC3930331

[82] Yeh JR, Zhang X, Nagano MC (2011) Wnt5a is a cell-extrinsic factor that supports self-renewal of mouse spermatogonial stem cells. Journal of Cell Science 124: 2357–2366. 21693582 10.1242/jcs.080903

[83] Niehrs C (2012) The complex world of WNT receptor signalling. Nat Rev Mol Cell Biol 13: 767–779. 23151663 10.1038/nrm3470

[84] Van Amerongen R, Mikels A, Nusse R (2008) Alternative wnt signaling is initiated by distinct receptors. Sci Signal 1: re9. 18765832 10.1126/scisignal.135re9

[85] Plaks V, Brenot A, Lawson DA, Linnemann JR, Van Kappel EC, et al (2013) Lgr5-Expressing Cells Are Sufficient and Necessary for Postnatal Mammary Gland Organogenesis. Cell Rep 3: 70–78. 23352663 10.1016/j.celrep.2012.12.017 PMC3563842

[86] Alexander CM, Goel S, Fakhraldeen SA, Kim S (2012) Wnt Signaling in Mammary Glands: Plastic Cell Fates and Combinatorial Signaling. Cold Spring Harbor perspectives in biology 4: a008037. 22661590 10.1101/cshperspect.a008037 PMC3475175

[87] Marjanovic ND, Weinberg RA, Chaffer CL (2013) Cell Plasticity and Heterogeneity in Cancer. Clinical Chemistry 59: 168–179. 23220226 10.1373/clinchem.2012.184655 PMC6220421

