# Supplementary figures and images for: Both Canonical and Non-Canonical Wnt Signaling Independently Promote Stem Cell Growth in Mammospheres
Source: PLoS One. 2014 Jul 14;9(7):e101800. doi: 10.1371/journal.pone.0101800 (PMC4096729; doi:10.1371/journal.pone.0101800)

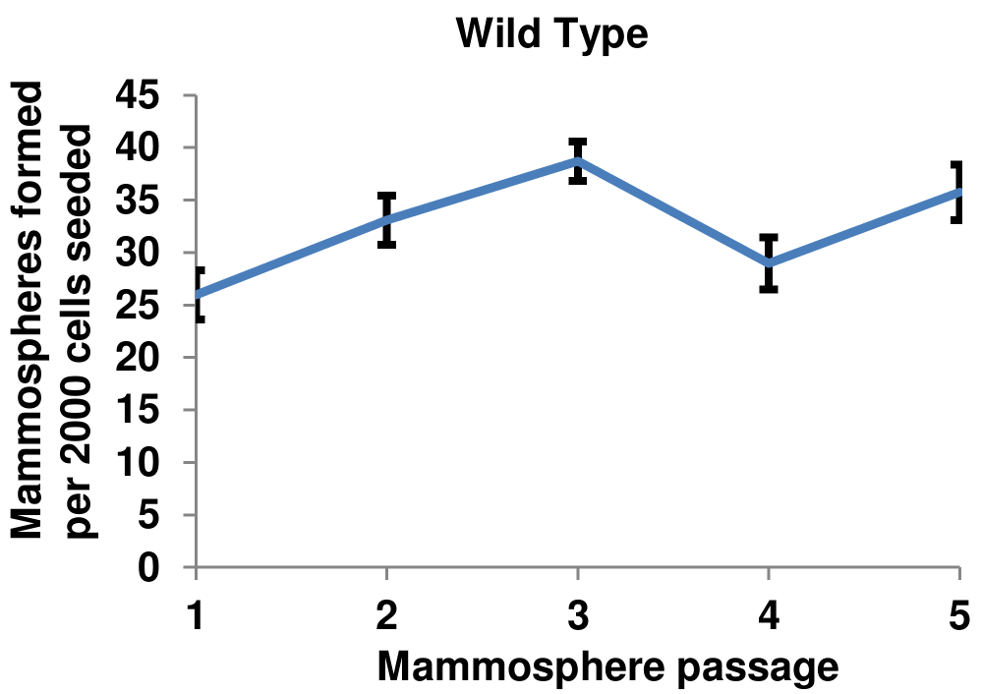

Supplement: Figure S1 — Wild-type mammospheres can be serially passaged for multiple generations. Wild-type mammosphere cultures were serially passaged weekly. The number of mammospheres formed per 2000 cells plated was assayed at each passage. Passage one represents the number of secondary mammospheres resulting from passage from primary to secondary culture. Error bars show 95% confidence intervals. (TIF) [file pone.0101800.s001.tif]
